# Corrigendum

**DOI:** 10.1002/jcla.24092

**Published:** 2021-11-24

In Volume 35, issue 4, 2021, in the article “Association of *ficolin‐1*, *ficolin‐3* gene variation and pulmonary tuberculosis susceptibility in a Chinese population” by Ye Li, En‐Qing You, Wen‐Hong Lin, Xiao‐Ning Liu, De‐Pei Shen, Xin‐Li Zhang, Dong‐Chun Ma, and Hong‐Miao Li., the second author name was incorrect.

The correct author is Qing‐hai You.

